# The ZFP36 Family as Post-Transcriptional Regulators in Physiology and Disease

**DOI:** 10.3390/ijms27146378

**Published:** 2026-07-17

**Authors:** Yuxuan Wen, Lichao Peng, Jing Wang

**Affiliations:** 1 The Queen Mary School, Jiangxi Medical College, Nanchang University, 999 Xuefu Road, Nanchang 330031, China; jp4217122152@qmul.ac.uk; 2 School of Basic Medical Sciences, Jiangxi Medical College, Nanchang University, 999 Xuefu Road, Nanchang 330031, China

**Keywords:** ZFP36 family, post-transcriptional regulation, mRNA decay, inflammation, tumorigenesis, therapeutic targets

## Abstract

The ZFP36 family proteins (TTP, ZFP36L1, and ZFP36L2) are RNA-binding proteins that function as key post-transcriptional regulators of gene expression. They bind AU-rich elements (AREs) in target mRNA 3′UTRs, recruit the CCR4-NOT deadenylation complex to trigger mRNA decay, and maintain homeostasis in immunity, barrier function, and stem cell fate. Rather than acting on single targets, all family members share a conserved mRNA destabilization mechanism, with outcomes determined by member-specific expression, kinase-mediated regulation, and cell-type-dependent target availability. Dysregulation of this network stabilizes mRNAs encoding pro-inflammatory cytokines, immune checkpoints, and oncogenes, driving pathogenesis of inflammation, autoimmunity, cancer, cardiovascular and neurodegenerative diseases. Individual family members exert context-dependent and sometimes opposing effects, so their net function depends on the specific cellular and disease context. Therapeutic strategies targeting ZFP36 activity, including phosphatase agonism and epigenetic modulation, have shown promising preclinical results, but clinical translation remains early. This review summarizes the molecular regulatory networks of the ZFP36 family and their physiological and pathological roles, emphasizing the mechanistic principles that unify family-member function and the contextual factors that diversify it, to provide a foundation for future therapeutic development.

## 1. Introduction and Core Mechanisms

### 1.1. Overview of the ZFP36 Family: Post-Transcriptional Trans-Acting Regulators

The stability and turnover of messenger RNA (mRNA) are key rate-limiting steps in determining cellular protein abundance. For genes requiring rapid environmental responsiveness—such as cytokines, proto-oncogenes, and early response genes encoding growth factors—their transient expression is primarily controlled by AU-rich elements (AREs) located in the 3′ untranslated regions (3′-UTRs) of mRNAs [1]. The ZFP36 family proteins act as core trans-acting factors that specifically recognize these cis-acting elements, forming a prototype of post-transcriptional regulatory mechanisms [2].

The mammalian ZFP36 family contains three members: tristetraprolin (TTP, gene name ZFP36), ZFP36L1, and ZFP36L2 [1,3]—that are functionally conserved tandem CCCH-type zinc finger proteins. These members each contain two RNA-binding zinc finger domains [2]. ZFP36 proteins link upstream signals (e.g., p38 MAPK) to downstream mRNA degradation machinery, including the CCR4-NOT complex. By binding the UUAUUUAUU motif in target mRNAs, they recruit deadenylases to initiate degradation, post-transcriptionally regulating immunity, stem cell fate, and tissue homeostasis [3,4,5].

All three family members share a common ARE-dependent mRNA decay mechanism, but their functions diverge via distinct expression patterns, kinase regulation, and non-conserved interaction regions. Physiologically, this enables them act as homeostatic brakes on immune activation, barrier function, and stem cell proliferation; in disease, the same machinery fails through recurring routes: lost mRNA surveillance, kinase-mediated silencing, and context-dependent target selection. These shared principles, more than the individual disease examples, define the feasibility and limitations of therapeutic targeting of this family [2].

### 1.2. Structural Basis: Conservation and Heterogeneity of Family Members

ZFP36 proteins share a conserved tandem CCCH zinc finger domain (TZF), containing two linked CX8CX5CX3H zinc finger motifs, each stabilized by zinc coordination [1]. Structural analyses have shown that these two zinc finger modules are connected by a short peptide linker and fold cooperatively into a rigid hydrophobic platform that binds single-stranded RNA [1,6]. The TZFs from TTP, ZFP36L1 and ZFP36L2 share >70% amino acid homology and have nearly identical in vitro RNA-binding properties, explaining their functional redundancy [1,2]. All three proteins also contain a conserved C-terminal CNOT1-binding motif (CIM) that specifically binds the CCR4-NOT complex core scaffold CNOT1 [7,8,9].

However, beyond the structured TZF core, their N- and C-terminal regions are evolutionarily divergent [2,4]. These flanking regions are largely intrinsically disordered (IDRs) and differ substantially in length and composition. Although this heterogeneity does not affect RNA recognition, it determines their distinct protein interaction networks, subcellular localization, and post-translational modifications. For example, all three rely on CRM1-mediated nuclear export, but their nuclear export signals (NESs) are located differently: TTP’s functional NES is located in the N-terminal, while ZFP36L1 and ZFP36L2 use a conserved C-terminal NES [10].

These divergent regions also specify member-specific interaction networks. TTP contains a unique N-terminal polyproline motif (PPPPGF) that anchors the 4EHP-GYF2 translational repression complex, forming a 3′end–TTP–GYF2–4EHP–5′cap loop that transiently blocks translation before mRNA decay; this motif is absent in ZFP36L1 and ZFP36L2, whose effects rely almost entirely on the decay pathway [1,7]. Conversely, ZFP36L1 and ZFP36L2 carry C-terminal proline-rich regions that may recruit specific cofactors and confer functional specialization relative to TTP [1,4,11].

Divergent promoter regions further produce markedly distinct expression patterns: TTP is transiently induced to high levels in stimulated myeloid cells, whereas ZFP36L1 and ZFP36L2 maintain higher basal expression in the lymphohematopoietic system and during development [12,13]. The family thus follows a “conserved core, divergent periphery” organization—a near-identical TZF providing shared RNA-binding specificity and redundancy, while divergent disordered termini generate distinct interaction networks and modes of regulation. This structural divergence underlies the member-specific functions described below.

### 1.3. Molecular Mechanism: The Universal Machine for mRNA Degradation

Despite divergent tissue expression, the mRNA decay mechanism of ZFP36 proteins is evolutionarily highly conserved. ZFP36 proteins function as molecular adapters linking target mRNAs to the cellular degradation machinery. Upon binding target mRNA 3′-UTRs, the global landscape of direct ZFP36 target mRNAs was first systematically delineated by PAR-CLIP, which identified hundreds of ARE-containing transcripts bound by ZFP36 across cell types [14]. They recruit the CCR4-NOT deadenylase complex via their C-terminal effector domain. This recruitment occurs mainly via direct interaction between the conserved ZFP36 C-terminal CNOT1-binding motif and the CCR4-NOT scaffold protein CNOT1 [1,15]. CCR4-NOT recruitment is the key initiating step of mRNA silencing. The complex first catalyzes rapid poly(A) tail removal, triggering two major degradation pathways: 3′→5′ degradation by the RNA exosome, and 5′→3′ degradation by XRN1 after DCP1/2-mediated decapping [1,15].

### 1.4. Dynamic Activity Switch: Phosphorylation Regulation

ZFP36 family activity is controlled by post-translational modifications that prevent excessive mRNA degradation. Key regulatory pathways include the p38 MAPK/MK2 and ERK-RSK cascades [16,17,18]. During physiological inflammatory responses, the p38 MAPK-MK2 cascade dominantly controls TTP activity. Upon pro-inflammatory stimulation, activated MK2 specifically phosphorylates two conserved serine residues (S52 and S178 in mice, corresponding to S60 and S186 in humans) on TTP [17,19]. This phosphorylation promotes high-affinity binding of TTP to 14-3-3 dimeric partner proteins, which in turn masks TTP’s C-terminal effector domain, preventing recruitment of CCR4-NOT and sequestering TTP within cytoskeletal complexes. Consequently, TTP is excluded from mRNA decay sites (processing bodies, P-bodies), inhibiting its degradative activity. Beyond P-body exclusion, MK2-induced TTP:14-3-3 complexes block TTP association with stress granules—cytoplasmic mRNA storage/triage foci—further uncoupling TTP from ARE-mRNA decay [20]. This leads to aberrant accumulation and sustained translation of pro-inflammatory transcripts (e.g., TNF-α) during acute inflammation [18,21]. Notably, MK2 similarly phosphorylates ZFP36L1 at Ser54, Ser92, and Ser203, suppressing its mRNA decay function [22].

In contrast, ZFP36L1 and ZFP36L2 are predominantly regulated by the ERK-RSK signaling pathway rather than MK2 under broad physiological contexts [18]. ERK and its downstream kinase RSK phosphorylate ZFP36L1 at Ser334 and ZFP36L2 at Ser492/494 [18,22]. These phosphorylations disrupt the interaction between ZFP36L1’s C-terminal CIM and CCR4-NOT scaffold CNOT1, blocking functional complex assembly. This impairs downstream decay enzyme recruitment, suppressing mRNA degradation [18]. This kinase specificity enables different ZFP36 members to respond to distinct upstream signals. Beyond MK2-mediated phosphorylation, TTP is subject to multi-layered regulation. Within this kinase axis, TTP phosphorylation is reversed by counteracting phosphatases such as PP2A and DUSP1, which restore its mRNA-decay activity [23,24]. Transcriptionally, ZFP36 acts as an immediate-early gene rapidly induced by various stimuli, including glucocorticoids [25]. Post-transcriptionally, TTP restricts its own expression via an ARE-dependent autoregulatory loop [26]. At the post-translational level, beyond activity masking, the phosphorylation state also dictates TTP protein stability: dephosphorylated TTP is rapidly degraded by the proteasome, whereas the phosphorylated form is relatively stabilized [27]. These integrated mechanisms tightly control the net pool of active TTP.

### 1.5. Tissue-Specific Expression and Functional Division

Although their mechanistic principles are conserved, functional specialization among family members is primarily determined by tissue-specific expression patterns. TTP is a prototypical early response gene transiently highly expressed in stimulated myeloid cells, where it chiefly governs control of acute inflammatory cytokines. TTP deficiency leads to systemic inflammatory syndrome [2,28,29]. By contrast, ZFP36L1 and ZFP36L2 are more stably expressed primarily in lymphoid and hematopoietic stem cells [11,30]. ZFP36L2 deletion causes hematopoietic stem cell (HSC) exhaustion [12]; combined deletion of ZFP36L1 and ZFP36L2 causes thymic developmental arrest and T-cell leukemia [11]. Additionally, ZFP36L1 shows unique expression in erythroid differentiation and vascular endothelium, regulating specific targets such as Stat5b [13] and Vegfa (vascular endothelial growth factor A) [31]. Under strong stimulation, family members may display compensatory synergy. Snyder et al. showed that triple knockout of TTP, ZFP36L1 and ZFP36L2 causes aberrant stabilization of multiple pro-inflammatory cytokine and chemokine mRNAs, triggering systemic inflammation and severe arthritis [28]. Thus, the three ZFP36 proteins are partially redundant in restraining myeloid inflammation.

These members share a near-identical RNA-binding core yet diverge in expression, dominant kinase, and terminal interaction motifs (Table 1), which underlies the cell-type-specific functions discussed below.

**Table 1:** Comparison of the three ZFP36 family members across their defining functional axes.

Feature	TTP (ZFP36)	ZFP36L1	ZFP36L2
Expression	Inducible; transient in stimulated myeloid cells [12,13]	Constitutive; lymphoid, endothelial, erythroid [11,13,30,31]	Constitutive; HSC, lymphoid, developmental [11,12,30]
Dominant kinase	p38 MAPK/MK2 [16,17]	ERK-RSK (also MK2) [18,22]	ERK-RSK [18]
Distinctive structure	N-terminal polyproline (4EHP-GYF2) motif [1,7]	C-terminal proline-rich region [1,4,11]	C-terminal proline-rich region [1,4,11]
Principal role	Restraint of acute inflammatory cytokines [2,28]	Cell quiescence; MZ B-cell identity; erythroid control [32,33,34]	HSC maintenance; memory T-cell quiescence; Treg function [12,35,36]

Note. TTP, tristetraprolin (ZFP36); HSC, hematopoietic stem cell; MZ, marginal zone; Treg, regulatory T cell. Phosphosites are shown in Figure 1A. Features and citations are detailed in Section 1.2, Section 1.3, Section 1.4 and Section 1.5 (and Section 2 for physiological roles).

**Figure 1:**
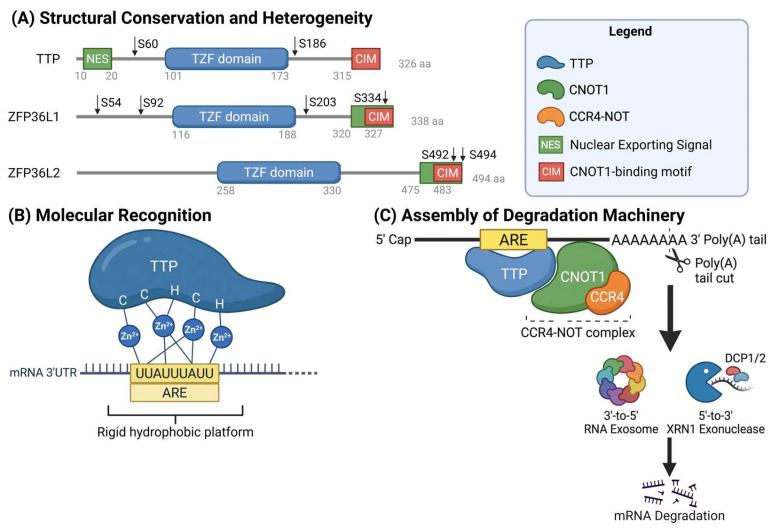
Structural features of the ZFP36 family proteins and mRNA degradation mechanism. (**A**) Structural Conservation and Heterogeneity: Displays the structural components of TTP, ZFP36L1, and ZFP36L2. All members contain a tandem zinc finger domain (TZF). The legend indicates the Nuclear Export Signal (NES) with a green box and the CNOT1-binding motif with a red box. Phosphorylation sites are marked for each protein: TTP (S60, S186), ZFP36L1 (S54, S92, S203, S334), and ZFP36L2 (S492, S494). (**B**) Molecular Recognition: Shows the TTP protein binding to the AU-rich element (ARE, indicated by the sequence UUAUUUAUU) on the mRNA 3′ untranslated region (3′UTR) via a rigid hydrophobic platform containing zinc ions (Zn^2+^) and C, C, H, C, H residues. (**C**) Assembly of Degradation Machinery: Depicts TTP recruiting the CCR4-NOT complex (containing CNOT1 and CCR4) after binding to the ARE. The diagram illustrates the cleavage of the 3′ Poly(A) tail (Poly(A) tail cut) and the subsequent two degradation pathways: 3′-to-5′ degradation executed by the RNA Exosome, and 5′-to-3′ degradation executed by the XRN1 Exonuclease following 5′ Cap removal by the DCP1/2 complex. Created in BioRender. koso, T. (2026) https://BioRender.com/8s1ir0f.

## 2. The Role of the ZFP36 Family in Physiological Development and Homeostasis

**Figure 2:**
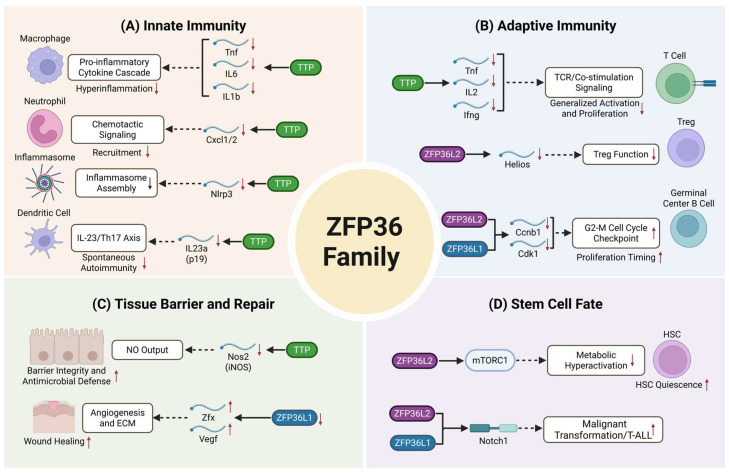
Hub-and-spoke network of the ZFP36 family: from mRNA targets to physiological phenotypes. The family acts as a central post-transcriptional hub controlling cell-type-specific programs. (**A**) Innate immunity: TTP downregulates early-response mRNAs (TNF, IL-6, IL-1β), Cxcl1/2, Nlrp3 and Il23a (p19), restraining hyperinflammation, chemotaxis, inflammasome assembly and spontaneous autoimmunity. (**B**) Adaptive immunity: TTP restricts conventional T-cell activation; ZFP36L2 tunes induced Treg function via Ikzf2 (Helios), and combined ZFP36L1/L2 deletion abolishes Treg suppression. In germinal center B cells, ZFP36L1/L2 cooperatively downregulate Ccnb1/Cdk1 to enforce the G2-M checkpoint. (**C**) Tissue barrier and repair: TTP downregulates Nos2 (iNOS) for barrier defense; ZFP36L1 represses Zfx and Vegf at steady state, and its injury-induced downregulation relieves repression to promote angiogenesis, ECM remodeling and wound healing. (**D**) Stem cell fate: ZFP36L2 downregulates mTORC1 to promote HSC quiescence; in lymphoid progenitors, ZFP36L1/L2 cooperatively restrict Notch1 to prevent T-ALL. Solid arrows: direct post-transcriptional regulation of target mRNAs; dashed arrows: downstream signaling and phenotypic outcomes. Red upward/downward arrows indicate upregulation/downregulation, respectively. Created in BioRender. koso, T. (2026) https://BioRender.com/enp76we.

Physiological homeostasis requires rapid pathogen responses and tissue regeneration, while preventing inappropriate resting activation. By post-transcriptionally regulating mRNA stability, ZFP36 family proteins maintain this balance in immune responses, tissue barrier function, and stem cell fate. At the molecular level, this means each member binds ARE-containing transcripts, recruits CCR4-NOT to initiate deadenylation, and thereby reduces protein output—blocking downstream signaling cascades that would otherwise drive inflammation, proliferation, or tissue remodeling. Consistent with their structural and regulatory divergence, the three members are not functionally interchangeable: TTP predominates in innate immune restraint, while ZFP36L1 and ZFP36L2 play primary roles in lymphocyte development, tissue barrier function, and stem cell maintenance. Below, we describe this division of labor and the cooperative redundancy observed under strong stimulation (Figure 2, Table 2) [28].

### 2.1. The “Quiescence” and “Braking” of the Immune System

#### 2.1.1. Threshold Control in Innate Immunity

As the first line of defense, innate immune cells rely on TTP to set a post-transcriptional threshold for inflammatory gene expression, preventing excessive activation by weak stimuli. In macrophages, TTP is the rate-limiting regulator of tumor necrosis factor-α (TNF-α), recruiting CCR4-NOT to degrade its mRNA and preventing systemic inflammatory syndrome [37]; it similarly restrains interleukin-6 (Il6) [38] and interleukin-1β (Il1b) [39] to calibrate the magnitude of the response. Beyond these core cytokines, TTP regulates multiple additional innate immune pathways: limiting dendritic cell maturation by degrading Tnf [43] and the IL-23 subunit Il23a/p19 [44], fine-tunes neutrophil recruitment through Cxcl1/Cxcl2 [40], restricts inflammasome assembly via interaction lncOlfr29 [41], and shapes immune tolerance by degrading the metabolic enzyme Ido1 [43]. Il23a/p19 regulation is particularly critical, as it prevents spontaneous colitis and arthritis [44]. Thus, TTP’s ARE-mediated decay does not merely suppress individual mediators but blocks the entire inflammatory cascade—from cytokine release through chemotaxis to immune-cell activation [40].

#### 2.1.2. Activation and Differentiation of T Lymphocytes

Unlike innate immunity, which depends predominantly on TTP, adaptive immune regulation relies on coordinated division of labor among ZFP36 family members. In conventional T cells, the family constrains activation thresholds and reinforces CD28 co-stimulation dependence [30,46,47]; TTP directly degrades the effector-cytokine mRNAs Il2 [48], Ifng, and Tnf [46] to limit proliferation and effector function, curbs Th17 pathogenicity via Il17a degradation [49], and the Akt/PI3K axis antagonizes TTP activity, linking this restraint to T-cell metabolic reprogramming [63]. By contrast, ZFP36L1 and ZFP36L2 play dominant roles in cytotoxic and regulatory compartments, with precise temporal specialization. In CD8^+^ T cells, ZFP36L1 acts early to restrict cytotoxic differentiation via NF-κB [50], while ZFP36L2 acts later to prevent cytokine storm by Ifng suppression [51]. In memory T cells, ZFP36L2 binds AREs in preformed cytokine mRNAs (e.g., Ifng) and blocks translation without degradation, maintaining a transcript reservoir for rapid recall while preventing cytokine leakage during quiescence [35]. In regulatory T cells, ZFP36L2 tunes induced Treg function via Helios (Ikzf2) mRNA degradation [36]; combined deletion of ZFP36L1 and ZFP36L2 abolishes Treg suppressive activity, causing fatal autoimmunity [52].

#### 2.1.3. Developmental Checkpoints in B Lymphocytes

ZFP36 family members function at multiple B-cell developmental stages. ZFP36L1 is essential for marginal zone (MZ) B-cell identity maintenance; its loss upregulates key transcription factors (e.g., Klf2, Irf8), causing MZ B-cell pool depletion [32]. During the germinal center response, ZFP36L1 and ZFP36L2 function cooperatively to enforce the G2-M checkpoint prior to mitosis via Ccnb1 and Cdk1 mRNA degradation [33], illustrating family member-specific division of labor. During terminal differentiation, ZFP36L1 negatively regulates plasma cell differentiation via Blimp1 (Prdm1) mRNA targeting [53]. Additionally, TTP regulates HLA-DQ mRNA turnover in B cells, modulating antigen presentation efficiency [64]. During malignant transformation, TTP suppresses B-cell lymphoma development via a c-Myc negative feedback loop [65], while ZFP36L1 regulates B-cell survival via Bcl2 mRNA modulation [66].

### 2.2. Defense and Repair of Tissue Barriers

#### 2.2.1. Dynamic Balance of the Intestinal Epithelium

In intestinal epithelial cells (IECs), TTP is a key factor in maintaining mucosal immune homeostasis. In acute injury models, IEC-specific TTP deletion causes aberrant stabilization of Nos2 (iNOS) mRNA; the resulting nitric oxide paradoxically protects by promoting mucus secretion and antibacterial defense [54]. Notably, this TTP function in IECs is highly autonomous and non-redundant; TTP-specific knockout does not trigger compensatory upregulation of its paralogs Zfp36l1 and Zfp36l2 [54]. Additionally, TTP indirectly modulates local Treg homeostasis via regulating Raldh2 (Aldh1a2) expression in intestinal dendritic cells, controlling retinoic acid synthesis [45]. During chronic inflammation, TTP functional loss is strongly associated with sustained Cox2 [67] and Il23 [44] expression, promoting colitis-associated cancer development.

#### 2.2.2. The Anti-Inflammatory Barrier of the Respiratory Epithelium

During lung pathogen invasion or injury, the ZFP36 family maintains immune balance by regulating inflammatory mediator mRNA stability and preventing excessive inflammatory damage. In airway epithelial cells, ZFP36L1 and ZFP36L2 downregulation contributes to asthma pathogenesis, and their re-expression significantly reduces Il6 and Il8 levels [55]. In acute lung injury (ALI), TTP limits excessive neutrophil pulmonary infiltration via Cxcl1 and Cxcl2 mRNA degradation [56]. In cigarette smoke-induced chronic obstructive pulmonary disease (COPD), impaired TTP expression sustains inflammation [57], while TTP functional enhancement effectively reduces pulmonary inflammation and airway remodeling [58]. Moreover, glucocorticoid anti-inflammatory effects partially depend on inducing TTP expression [68], while radiation injury promotes Tnf secretion via TTP phosphorylation and inactivation [69]. In lung ischemia–reperfusion injury, TTP also protects via the CREBBP pathway [70]. Across these conditions, a common mechanism emerges: ZFP36-mediated degradation of chemokine/cytokine transcripts interrupts the positive feedback loop where immune cell recruitment further amplifies inflammation.

#### 2.2.3. Skin Barrier and Wound Healing

In keratinocytes, TTP regulates both inflammatory skin diseases (e.g., psoriasis) and wound healing. At homeostasis, TTP prevents spontaneous skin inflammation by Tnf [59] and Il17a [49] mRNA degradation. In psoriatic lesions, ZFP36 proteins mediate dermal fibroblast inflammatory phenotypes via Il6 and Cxcl8 regulation [71]. During wound re-epithelialization, miR-93-3p specifically downregulates Zfp36l1 [60]. This ZFP36L1 downregulation relieves Zfx transcriptional repression, enabling keratinocytes to initiate proliferative and migratory programs to accelerate wound closure [60]. In addition, ZFP36L1 is growth factor-regulated and modulates keratinocyte Vegf production [31], while TTP prevents skin carcinogenesis via Areg-mediated EGFR signaling regulation [61].

### 2.3. Stem Cell Fate Determination and Differentiation

#### 2.3.1. Hierarchical Regulation of Hematopoietic Stem Cells

At the top of the hematopoietic hierarchy, ZFP36L2 is critical for maintaining HSC pool integrity. Complete Zfp36l2 deletion causes lethal anemia and hemorrhage in mice via hematopoietic failure [12]. ZFP36L2 potently suppresses mTORC1 signaling to prevent metabolic decompensation [72]; as mTORC1 activation drives HSC exhaustion, this post-transcriptional metabolic regulation is critical for preserving stem cell quiescence. Moreover, HSC self-renewal is regulated by multiple coordinated mechanisms, including ZFP36L2-mediated RNA stability control and endoplasmic reticulum-associated degradation (ERAD), which constitute key components of the quiescence barrier [62]. By contrast, ZFP36L1 negatively regulates specific erythroid differentiation; its deletion causes abnormal erythroid progenitor expansion [13]. In myeloid cells, ZFP36 proteins cooperatively regulate inflammatory responses [28].

#### 2.3.2. Development of Lymphoid Progenitors

During the early stages of lymphocyte development, ZFP36L1 and ZFP36L2 exhibit marked functional synergy, together forming a barrier against malignant transformation of progenitor cells [4]. In thymic T-cell development, combined loss of Zfp36l1 and Zfp36l2 results in dysregulated expression of Notch1 and its downstream target genes [11]. This dysregulation disrupts cell cycle control, causing genomic instability and ultimately T-cell acute lymphoblastic leukemia (T-ALL) [11].

#### 2.3.3. Other Tissue Stem Cells and Aging

The ZFP36 family also regulates other stem cell populations. In adipose-derived mesenchymal stem cells, TTP promotes osteogenic differentiation by degrading Klf3 mRNA [73], critical for skeletal homeostasis. During aging, MDSC expansion drives immunosenescence, and TTP limits aberrant MDSC accumulation [74]. In mammary progenitor cells, TTP maintains cell survival via Tnf regulation, critical for mammary tissue regenerative capacity [75]. During neural stem cell neuronal differentiation, ZFP36L1 downregulation is also essential [76].

## 3. Dysregulation of the ZFP36 Family and Pathological Mechanisms

Under physiological conditions, the ZFP36 family is critical for immune homeostasis. However, functional abnormalities including expression loss from genetic defects or epigenetic silencing, and impaired activity from aberrant post-translational modifications, disrupt post-transcriptional regulation [27,77,78]. Loss of this negative feedback aberrantly stabilizes mRNAs encoding pro-inflammatory cytokines, chemokines, growth factors and immune checkpoints, creating a chronic inflammation positive feedback loop that drives tumor progression and immune evasion [79]. This loop closes at the molecular level: stabilized cytokine mRNAs produce more protein output, further activating p38 MAPK/MK2 to phosphorylate and inactivate remaining ZFP36, completing a self-reinforcing cycle. Importantly, the three failure routes—genetic deletion, epigenetic silencing, and kinase-mediated inactivation—converge on the same molecular outcome: loss of ARE-mediated mRNA surveillance, with cell-type-dependent target availability determining whether the consequence is inflammation, autoimmunity, or cancer.

### 3.1. The Context-Dependent Role of the ZFP36 Family in Malignant Tumors

#### 3.1.1. The Tumor-Suppressive Baseline: Shared Post-Transcriptional Regulatory Principles

Across diverse human malignancies, loss of ZFP36 family activity is a recurrent, functionally important event. These proteins do not act through a single oncogenic target; they are post-transcriptional brakes on several cancer hallmarks at once, so their silencing has pleiotropic pro-tumorigenic effects.

In proliferation and survival, ZFP36 family members cap key oncogenic drivers. TTP loss derepresses AP-1/c-Jun [80] and c-Myc [65] in breast cancer and hematologic malignancies, accelerating G1–S transition via c-Jun/c-Myc-driven cyclin D1/E transactivation, while ZFP36L1 deficiency stabilizes the anti-apoptotic transcript BCL2 in B-cell malignancies [66]. In invasion and metastasis, the family restrains extracellular matrix (ECM) degradation and epithelial–mesenchymal transition (EMT): TTP targets MMP2 and MMP9 [81], the urokinase plasminogen activator system (PLAU and PLAUR) [82], and the EMT driver Snail1 [83], so its loss simultaneously dismantles ECM degradation and EMT transcriptional programming, removing both physical and transcriptional barriers to metastasis. In metabolic reprogramming and angiogenesis, TTP promotes decay of HIF-1α mRNA (Hif1a) [84]; under the hypoxia common in solid tumors, losing this checkpoint allows HIF-1α to accumulate, driving VEGFA-dependent angiogenesis [85] and a shift to aerobic glycolysis [86]. TTP also suppresses VEGFA independently of HIF-1α in colorectal cancer [85], giving it at least two anti-angiogenic routes. At the immune interface, TTP promotes decay of CD47 mRNA [87]; its loss in head and neck squamous cell carcinoma raises CD47, which engages SIRPα to impair macrophage clearance of tumor cells [87], while dysregulation of ZFP36L1 and ZFP36L2 may indirectly weaken anti-tumor T-cell responses [30].

These findings position the ZFP36 family as broad-spectrum tumor suppressors that limit proliferation, invasion, metabolic adaptation, and immune evasion; disease- and target-specific details are given in Table 3. This pattern is not universal: in some tumor types certain members show context-dependent, sometimes paradoxical behavior, discussed in Section 3.1.3.

#### 3.1.2. Tumor Microenvironment-Driven Mechanisms of Functional Silencing

**Figure 3:**
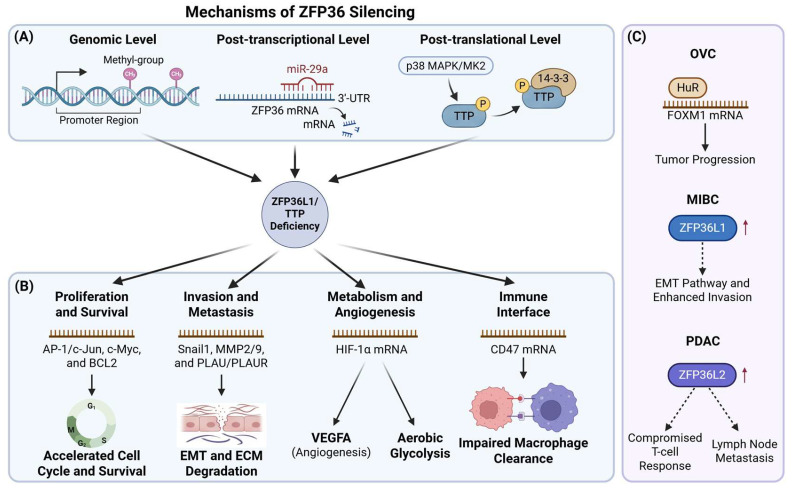
Molecular mechanisms of ZFP36 family dysregulation driving TME remodeling and tumor progression. (**A**) Three convergent routes silence ZFP36 family activity—promoter hypermethylation (genomic), miR-29a-mediated decay (post-transcriptional), and p38 MAPK/MK2 phosphorylation with 14-3-3 sequestration (post-translational)—producing ZFP36L1/TTP deficiency. (**B**) This deficiency derepresses multiple cancer hallmarks: proliferation and survival, invasion and metastasis, metabolism and angiogenesis, and the tumor–immune interface, via the representative targets shown. (**C**) Context-dependent and paradoxical roles in ovarian cancer (OVC), muscle-invasive bladder cancer (MIBC), and pancreatic ductal adenocarcinoma (PDAC). Solid arrows: Direct post-transcriptional regulation of target mRNAs; dashed arrows: downstream signaling and phenotypic consequences. Red upward arrows indicate upregulation. Created in BioRender. Wen, Y. (2026) https://BioRender.com/avd6h1l.

Tumors can overcome ZFP36-mediated mRNA surveillance through three distinct, potentially synergistic routes, acting at the genomic, post-transcriptional, and post-translational levels (Figure 3).

The best-documented is epigenetic silencing through promoter hypermethylation of the ZFP36 locus, demonstrated in hepatocellular carcinoma [92], non-small cell lung cancer [78], and breast cancer. In colorectal cancer, HDAC-mediated chromatin compaction similarly represses TTP, and HDAC inhibitors restore it via EGR1-dependent induction [91], indicating this silencing is both widespread and potentially reversible. Post-transcriptionally, miR-29a targets the 3′-UTR of ZFP36 mRNA in invasive breast cancer, promoting its decay and facilitating EMT and metastasis [88]. The third route is functional silencing at the protein level, which can inactivate ZFP36 even when the protein is abundant: as in chronic inflammation (Section 3.2.1), sustained p38 MAPK/MK2 activity hyperphosphorylates TTP and promotes 14-3-3 binding [17], sequestering TTP in the cytoskeletal scaffold, excluding it from P-bodies, and preventing CCR4-NOT recruitment [114].

In malignant glioma, TTP is abundantly expressed yet heavily phosphorylated; a phosphorylation-deficient mutant repressed IL-8, VEGF and IL-6 more strongly than wild-type, with stronger antiproliferative effect [115]. As in chronic inflammation (Section 3.2.1), TTP can be inactivated by phosphorylation rather than lost by downregulation. Whether this extends to other solid tumors is untested. These routes need not be exclusive: promoter methylation, miR-29a and phosphorylation could converge on one tumor.

#### 3.1.3. Context-Dependent Roles: Mechanistic Drivers of the Oncogenic Switch

Although the tumor-suppressive role of the ZFP36 family is well established, it is not absolute. In a few tumor types, individual members have been linked to paradoxical outcomes such as enhanced invasiveness, stromal remodeling, or poor prognosis. In most cases the evidence is correlative, from transcriptomic profiling or clinical datasets, and the mechanisms are not experimentally established (summarized in Table 3).

In muscle-invasive bladder cancer (MIBC), ZFP36L1 appears to suppress tumor-cell self-renewal early, yet at advanced stages high ZFP36L1 expression is associated with EMT-pathway activation and greater invasiveness [93]; the responsible target mRNAs have not been identified. Hepatocellular carcinoma shows a parallel pattern: TTP promotes hepatic inflammation and tumor initiation early but restrains progression later [116]. In both, tumor stage can determine whether ZFP36 activity is net tumor-promoting or tumor-suppressive.

Pancreatic ductal adenocarcinoma (PDAC) is more sharply contested, with directly conflicting evidence. One study found ZFP36L2 overexpressed in PDAC specimens and repressed by the tumor-suppressive miRNA miR-375; high expression correlated with lymph-node metastasis and shorter survival, and knockdown reduced aggressiveness, leading the authors to propose an oncogenic role [94]. A more recent large-scale multiomic study reached the opposite conclusion, nominating ZFP36L2 as a loss-of-function tumor suppressor, since its overexpression suppressed proliferation in PDAC cell lines, consistent with ZFP36L2 in most other malignancies [95]. The two are hard to reconcile: the discrepancy may reflect differences in disease stage or cellular compartment, or the gap between correlative profiling and direct functional perturbation. PDAC thus remains a genuine open controversy rather than a settled oncogenic role.

Ovarian cancer (OVC) is different again: the issue is not that TTP becomes oncogenic, but that aggressive disease persists even when TTP is not overtly lost, because the decisive variable is which RNA-binding protein (RBP) dominates shared ARE targets. Here HuR is the active oncogenic driver. In high-grade serous carcinoma, HuR stabilizes pro-oncogenic transcripts such as FOXM1—normally restrained by SOCS7-mediated ubiquitination of HuR—and HuR overexpression correlates with metastasis and poor survival [96,97]. TTP itself is low in mesenchymal-type ovarian cells, and restoring it degrades the EMT inducers Twist1 and Snail1 to reverse invasion [98]. The ovarian paradox is therefore an HuR-dominated TTP/HuR imbalance (Section 4.3), a functional override of the kind described in Section 3.1.2 rather than a true reversal of TTP’s tumor-suppressive role.

These divergent outcomes are also consistent with ZFP36L1 and ZFP36L2 differing from TTP: they are constitutively expressed in lymphoid, hematopoietic, and developmental compartments rather than transiently induced in myeloid cells [12,13], and their non-conserved terminal regions give them distinct protein-interaction networks [1,2]. The family members are therefore unlikely to be interchangeable across tumor contexts, even though the specific molecular routes remain undefined.

In summary, whether a ZFP36 member promotes or impedes tumor progression depends on tumor stage, the prevailing target-mRNA repertoire, the member involved, and competition with other RBPs. These determinants are unevenly established, from mechanistically traceable (OVC) to unresolved (PDAC, MIBC), and the associations above should be read with caution pending functional studies in stage-appropriate models.

### 3.2. Roles of the ZFP36 Family in Non-Cancer Diseases

#### 3.2.1. Chronic Inflammation and Autoimmune Diseases

**Figure 4:**
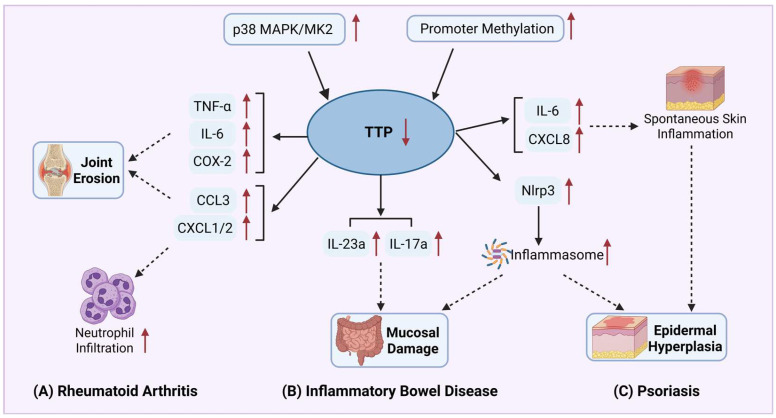
Pathological mechanisms of TTP dysregulation in chronic inflammatory and autoimmune diseases. TTP functional inactivation relieves post-transcriptional repression of its pro-inflammatory targets, driving multiple autoimmune diseases. (**A**) Rheumatoid arthritis (RA): Silenced TTP derepresses TNF-α, IL-6 and COX-2, promoting joint destruction; accumulated CCL3 and CXCL1/2 drive synovial neutrophil infiltration and further joint erosion. (**B**) Inflammatory bowel disease (IBD): TTP functional loss elevates IL-23α and IL-17α, activating the pathogenic IL-23/IL-17 axis; upregulated NLRP3 promotes inflammasome assembly, collectively driving mucosal damage. (**C**) Psoriasis: TTP downregulation raises IL-6 and CXCL8 and, together with NLRP3 inflammasome activation, drives epidermal hyperplasia and psoriatic lesions. Throughout, the elevated but functionally silenced state of TTP typical of chronic disease is distinguished from simple loss of expression. Solid arrows: Direct molecular regulation; dashed arrows: downstream pathological consequences. Red upward/downward arrows indicate upregulation/downregulation, respectively. Created in BioRender. koso, T. (2026) https://BioRender.com/tcagygb.

In chronic inflammatory and autoimmune diseases, TTP abundance and function are often uncoupled: TTP protein is often abundant or even elevated in diseased tissue but fails to resolve inflammation. Thus, its regulatory output is determined not by expression level but by post-translational modification state, subcellular localization, and competition with other RBPs. Kinase-mediated functional silencing, spatially restricted chemokine control, and barrier-specific dysregulation each contribute to sustained inflammation, as described below (Figure 4).

##### Kinase-Mediated Functional Silencing of ZFP36 and Persistent Inflammation

In chronic inflammatory diseases (e.g., rheumatoid arthritis, RA), TTP exhibits a unique “functional silencing” state. Unlike rapid TTP activity recovery during acute inflammation resolution, TTP mRNA and protein levels are significantly elevated in RA patient synovial fibroblasts and infiltrating macrophages [99], yet this increase fails to produce an effective anti-inflammatory response. This functional silencing is primarily caused by persistent activation of the p38 MAPK/MK2 signaling pathway, resulting in constitutive hyperphosphorylation of TTP at critical serine residues [16,17]. Phosphorylated TTP tightly binds chaperone 14-3-3 and is sequestered in cytoskeletal scaffolds [17], spatially blocking CCR4-NOT deadenylase complex recruitment [114]. Consequently, TTP loses its ability to degrade key proinflammatory targets such as TNF-α [37], IL-6 [38], and COX-2 [67]. This phosphorylation-driven silencing also limits TTP tumor suppressor activity in solid tumor microenvironments (Section 3.1), indicating a shared pathological mechanism rather than a disease-specific anomaly. Consistent with this mechanism, the PP2A agonist FTY720 reverses TTP phosphorylation, restores its decay activity, and alleviates arthritis [21,23], while the phosphatase DUSP1 modulates this loop via a prostaglandin E2 network [24]. In diabetic nephropathy, TTP expression inversely correlates with proteinuria, suggesting a protective role in chronic renal inflammation [117]. As abundant TTP can be functionally inert, its expression level alone is an unreliable biomarker; therapeutic reactivation requires correcting its phosphorylation state rather than simply increasing expression (Section 4.1).

##### Chemokine Regulation and Local Tissue Immune Cell Infiltration

Beyond systemic cytokines, TTP selectively governs local immune cell infiltration. In TTP-deficient macrophages, aberrant CCL3 mRNA stabilization drives excessive neutrophil recruitment to joint synovium [100]. Consistent with this compartmentalization, blocking CCL3 reduces local bone erosion and synovitis [100] without correcting systemic cachexia or elevated plasma TNF [29], revealing a spatial dissociation between TTP-mediated local tissue damage and systemic inflammation that has direct therapeutic implications. This compartmentalized control extends to other settings: TTP restrains neutrophil recruitment via Cxcl1/Cxcl2 in fibroblasts [71], suppresses pulmonary neutrophil infiltration via non-hematopoietic cell restoration [56], and regulates radiation-induced TNF-α production in lung macrophages [69].

##### Dysregulation of Intestinal and Skin Barrier Immunity

In psoriasis and inflammatory bowel disease (IBD), loss of TTP function directly activates the pathogenic IL-23/IL-17 axis. Upstream, TTP tightly restricts Il23a (p19) mRNA stability in dendritic cells [44]; downstream, TTP degrades Il17a mRNA in Th17 cells [49]. Loss of this dual negative regulation causes excessive IL-23 production and Th17 effector function derepression, synergistically triggering inflammatory cascades [44,49]. Beyond this axis, TTP also limits inflammasome activity via non-ARE-dependent Nlrp3 mRNA degradation [39], a restraint relieved by lncRNA lncOlfr29 via TTP-Nlrp3 binding blockade [41]; additionally, TTP maintains epithelial barrier integrity via Nos2 regulation in intestinal cells [54]. Consistent with a causal role in human disease, ZFP36 family promoter methylation aggravates NLRP3-driven inflammation in psoriatic fibroblasts [102], and TTP phosphorylation status correlates with pediatric IBD activity [101].

#### 3.2.2. Cardiovascular Diseases

**Figure 5:**
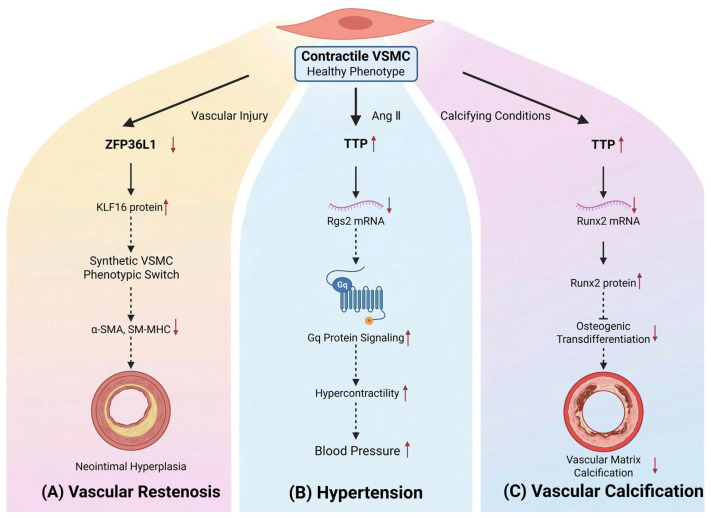
Regulatory roles of the ZFP36 family in VSMC phenotypic switching and cardiovascular disease. Distinct pathological stimuli alter ZFP36 family expression and function, driving divergent vascular outcomes from a healthy contractile VSMC baseline. (**A**) Vascular restenosis: Injury downregulates ZFP36L1, relieving repression of Klf16; the resulting rise in KLF16 drives a contractile-to-synthetic switch (loss of α-SMA, SM-MHC) that promotes neointimal hyperplasia. (**B**) Hypertension: Angiotensin II upregulates TTP, which degrades Rgs2 mRNA; loss of RGS2 unleashes Gq signaling, causing sustained VSMC hypercontractility and elevated blood pressure. (**C**) Vascular calcification: Upregulated TTP is protective, degrading Runx2 mRNA to limit VSMC osteogenic transdifferentiation and matrix calcification. Solid arrows, direct post-transcriptional regulation of target mRNAs; dashed arrows, downstream signaling and phenotypic consequences. Red upward/downward arrows indicate upregulation/downregulation, respectively. Created in BioRender. koso, T. (2026) https://BioRender.com/9xk41wp.

The cardiovascular system exemplifies that the net effect of individual ZFP36 family members is context-dependent. As detailed below, TTP is protective in some vascular cell types and conditions but pathogenic in others, demonstrating that ZFP36 function cannot be predicted by expression level alone and must be interpreted in a specific cellular and signaling context (Figure 5).

##### Vascular Smooth Muscle Cell Phenotypic Switching and Vascular Remodeling

Vascular smooth muscle cell (VSMC) phenotypic switching from contractile to synthetic state is central to atherosclerosis and restenosis. In injury-induced restenosis, transcription factor KLF16 overexpression drives excessive VSMC proliferation and migration, and ZFP36L1 is a key protective brake on this transition [103]: via KLF16 mRNA degradation, it restores contractile markers (α-SMA, SM-MHC) and limits neointimal hyperplasia [103]. Consistent with a protective role for the family, smooth-muscle-specific TTP deletion also exacerbates neointimal proliferation [104]. TTP additionally guards against vascular calcification: under calcifying conditions, upstream factors such as the adipokine CTRP13 sustain TTP activity by inhibiting its phosphorylation, and active TTP degrades Runx2 mRNA to suppress VSMC osteogenic transdifferentiation [105]. Similarly, in adipose-derived mesenchymal stem cells, TTP targets KLF3 to restrain ectopic ossification [73].

##### Disruption of Vascular Tension Homeostasis and Abnormal Blood Pressure Regulation

Normal blood pressure regulation depends on balanced vascular tone, maintained by endothelial cells (vasodilation) and VSMCs (vasoconstriction), where TTP exerts pronounced cell-type-specific effects. In endothelial cells, TTP primarily mediates vascular protection and vasodilation via degrading mRNAs encoding adhesion molecules (e.g., E-selectin, ICAM-1, VCAM-1), preventing pathological monocyte adhesion and vascular inflammation [118]. Concurrently, TTP suppresses oxidative enzyme Nox2, preserving endothelial nitric oxide synthase (eNOS) function and normal vasodilatory capacity [119]. By contrast, in VSMCs, TTP promotes hypertension progression. In angiotensin II (Ang II)-induced hypertension models, TTP expression in VSMCs is aberrantly upregulated, accelerating Rgs2 mRNA degradation [106]. As RGS2 is a key negative regulator of Gq protein-mediated vasoconstrictive signaling [107], its depletion relieves this inhibition, exacerbating contractile signaling [108]. Endothelial vasodilation impairment combined with VSMC contractile hyperactivity collectively causes aberrant vascular tone elevation and sustained hypertension [106].

#### 3.2.3. Neurological Diseases

**Figure 6:**
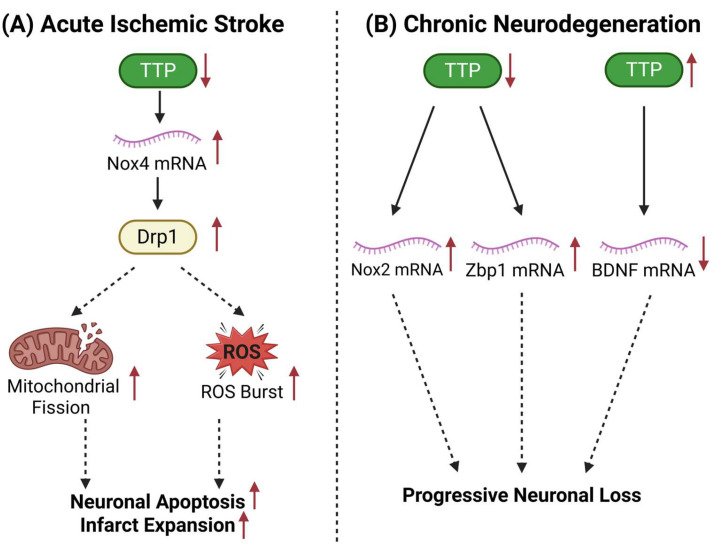
Regulatory roles of TTP in neurological disease. TTP dysregulation is associated with distinct pathological cascades in acute brain injury and chronic neurodegenerative disease. (**A**) Acute ischemic stroke: TTP downregulation relieves Nox4 mRNA post-transcriptional repression; increased NOX4 promotes Drp1-mediated mitochondrial fission and ROS burst, driving neuronal apoptosis and infarct expansion. (**B**) Chronic neurodegeneration: TTP exhibits context-dependent bidirectional dysregulation. TTP deficiency stabilizes Nox2 mRNA (exacerbating Parkinson’s disease oxidative stress) and Zbp1 mRNA (promoting Alzheimer’s disease microglial inflammation); conversely, aberrant TTP upregulation accelerates brain-derived neurotrophic factor (Bdnf) mRNA degradation, reducing neurotrophic support. These processes collectively drive progressive neuronal loss. Solid arrows: Direct post-transcriptional regulation of target mRNAs; dashed arrows: downstream cellular and pathological consequences. Red upward/downward arrows indicate upregulation/downregulation, respectively. Created in BioRender. koso, T. (2026) https://BioRender.com/droxdh6.

In neurological disorders, ZFP36 family-mediated post-transcriptional regulation is highly context-dependent, with TTP exerting divergent effects in acute versus chronic conditions (Figure 6). In acute brain injury, TTP primarily exerts neuroprotective effects via anti-inflammatory and anti-apoptotic pathways. During cerebral ischemia–reperfusion injury, TTP specifically binds the 3′-UTR of Nox4 mRNA, inhibiting NOX4-mediated activation of mitochondrial fission protein Drp1. This prevents excessive mitochondrial fragmentation and reactive oxygen species (ROS) bursts, protecting neurons from apoptosis [109]. In intracerebral hemorrhage models, TTP potently suppresses inflammation and significantly downregulates pro-apoptotic protein caspase-3, reducing secondary cerebral edema and tissue water content, ultimately improving neurological function [120].

In chronic neurodegenerative diseases, TTP regulatory networks are more complex. In Parkinson’s disease (PD), TTP exerts protective effects via Nox2 mRNA destabilization, blocking oxidative stress cascades and safeguarding dopaminergic neurons [110]. Conversely, in Alzheimer’s disease (AD), TTP exerts opposing effects: as a compensatory neuroprotective mechanism, TTP degrades ZBP1 transcripts to inhibit microglial NLRP3 inflammasome activation, exerting anti-inflammatory functions [111]. However, abnormally elevated TTP expression within brain tissue also contributes to pathological dysregulation. TTP binds and accelerates brain-derived neurotrophic factor (BDNF) mRNA degradation [112]. Clinical analyses show that TTP and miR-16 upregulation in AD patient temporal cortex correlates with reduced BDNF levels [113], although TTP’s causal role in this reduction remains to be confirmed by functional studies. Simultaneously, downregulated ROQUIN expression in this region further indicates a multifactorial dysregulation network, exacerbating central nervous system inflammation and cognitive decline [113].

The coexistence of protective and detrimental TTP activities in a single disease further demonstrates that the net effect of ZFP36 family members is determined not by expression level but by the predominant target transcripts in a specific cellular and disease context. Additionally, the ZFP36 family is critical for neuronal cell fate plasticity. During astrocyte-to-neuron reprogramming, ZFP36L1 downregulation is a critical step, driven specifically by miR-124 targeting [76].

## 4. Therapeutic Strategies and Translational Challenges Targeting the ZFP36 Family

**Figure 7:**
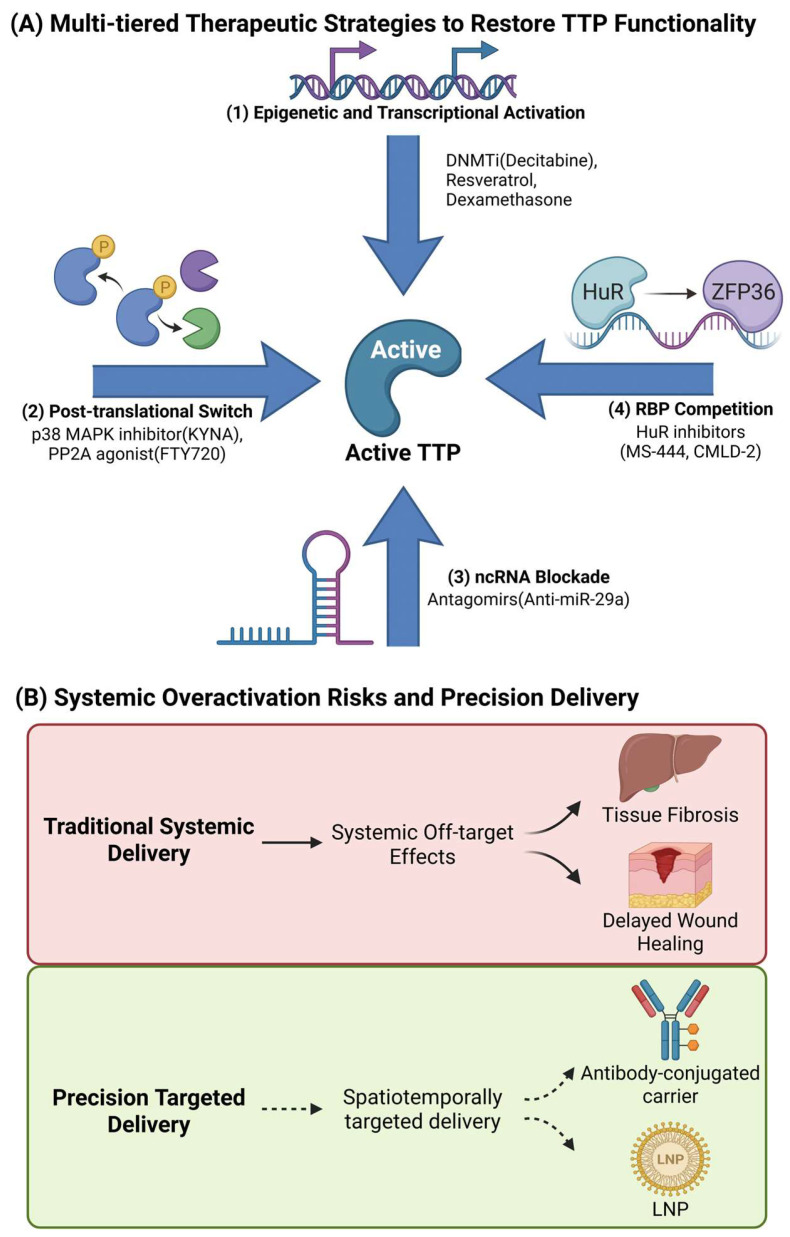
Multi-tiered pharmacological interventions targeting the TTP/ZFP36 family and translational challenges. (**A**) Four complementary strategies to restore TTP function: (1) epigenetic/transcriptional activation, (2) post-translational silencing reversal, (3) ncRNA blockade, and (4) RBP competition (representative agents shown). (**B**) Systemic overactivation risks and precision delivery. Non-specific systemic delivery poses safety risks via physiological tissue repair interference, causing off-target effects (e.g., tissue fibrosis and delayed wound healing); spatiotemporally targeted delivery systems (e.g., antibody-conjugated carriers or LNPs) are required to restrict modulation to pathological microenvironments. Panel A approaches are each supported by preclinical functional data (Section 4). Solid arrows: Established systemic-delivery risks; dashed arrows: conceptual delivery strategies not yet validated for ZFP36. No ZFP36-targeted agent has entered clinical trials. Created in BioRender. koso, T. (2026) https://BioRender.com/vcoue35.

Because these failure routes converge on a single molecular defect—loss of ARE-mediated mRNA surveillance—restoring ZFP36 function, whether by phosphatase agonism, epigenetic derepression, or expression enhancement, can suppress multiple downstream pathogenic cascades from one node. This convergence provides the mechanistic rationale for targeting the family therapeutically. The therapeutic strategies discussed below differ in their level of evidence. Some (e.g., phosphatase-based TTP reactivation and ZFP36 promoter epigenetic derepression) are supported by functional studies in cell and animal models, while others, particularly targeted delivery systems (Section 4.4), remain largely conceptual. Notably, to our knowledge, no ZFP36-targeted agent has entered clinical trials; thus, the strategies below represent future research directions rather than validated therapies (Figure 7).

### 4.1. Pharmacological Activation Based on Post-Translational Modifications

TTP activity is tightly regulated by post-translational modifications. The p38 MAPK/MK2 pathway is a key negative regulatory axis: MK2 phosphorylates TTP at Ser52/178 (mouse) or Ser60/186 (human), generating 14-3-3 docking sites. This 14-3-3 binding sequesters TTP in the cytoplasm, preventing target mRNA association and inhibiting its function [121]. Thus, pharmacological strategies to restore TTP function focus on disrupting kinase-phosphatase dynamic balance. Two complementary approaches are used: activating phosphatases to promote TTP dephosphorylation, and inhibiting kinases (e.g., MK2) to reduce TTP phosphorylation. This dual strategy restores TTP mRNA degradation activity, reestablishing its critical post-transcriptional regulation of inflammatory and oncogenic gene expression.

#### 4.1.1. Functional Restoration Mediated by Protein Phosphatase Agonists

Protein phosphatase 2A (PP2A) is the key enzyme responsible for removing phosphate groups from TTP, and reactivation of PP2A is essential for TTP’s return to P-bodies. Rahman et al. identified immunomodulatory drug FTY720 (Fingolimod) and its non-immunosuppressive derivative AAL(S) as allosteric agonists of PP2A [23]. In lung epithelial cell models, these compounds enhance PP2A activity, induce TTP dephosphorylation, and significantly accelerate pro-inflammatory mRNA degradation (e.g., Tnf, Il6) [23]. This approach directly targets TTP activation, bypassing complex upstream signaling crosstalk, and reverses hormone-resistant airway inflammation [19].

#### 4.1.2. Kinase Signal Pathway Inhibition and Metabolic Modulation

MK2 is the direct kinase that inactivates TTP. Blocking the p38 MAPK/MK2 pathway can maintain TTP activity. Patial et al. generated a TTPΔARE mouse model by deleting AREs in the TTP mRNA 3′-UTR, abolishing negative autoregulation and markedly elevating endogenous TTP protein levels [26]. This sustained TTP elevation conferred robust resistance to inflammatory pathologies (e.g., rheumatoid arthritis) [26]. Beyond synthetic inhibitors, endogenous metabolites also show regulatory potential. Walczak et al. reported that kynurenic acid (KYNA), a tryptophan metabolite, significantly inhibits p38 MAPK and PI3K/Akt pathway phosphorylation in colon cancer models [122]. This metabolic regulation indirectly sustains TTP in a non-phosphorylated active state by reducing downstream MK2 activation, thereby suppressing tumor proliferation and inflammatory microenvironment [122]. These findings suggest that pharmacological modulation of tryptophan metabolism (e.g., via IDO inhibitors) may restore TTP function and enhance its anti-inflammatory and immunomodulatory effects, although this requires direct experimental validation.

#### 4.1.3. Dual Synergistic Regulatory Mechanism of Glucocorticoids

Recent discoveries reveal that classical anti-inflammatory glucocorticoids (e.g., dexamethasone) exert effects closely linked to the ZFP36 family. Dexamethasone acts via two independent pathways: it rapidly induces mRNA transcription of TTP, ZFP36L1, and ZFP36L2 [25]; simultaneously, it strongly upregulates dual-specificity phosphatase 1 (DUSP1/MKP-1) to inhibit p38 MAPK activity [16,123]. This dual regulation elevates TTP protein abundance while preserving its mRNA degradation activity via DUSP1-mediated dephosphorylation [16]. This mechanism provides a therapeutic rationale for combining glucocorticoids with PP2A agonists in clinical interventions.

### 4.2. Gene Intervention Based on Epigenetic and Transcriptional Remodeling

In many malignancies and chronic disease states, targeting post-translational modifications alone (e.g., the dephosphorylation strategies described above) is insufficient to restore TTP function, as its encoding gene ZFP36 is often transcriptionally silenced. This silencing is primarily caused by promoter epigenetic modifications or non-coding RNA-mediated post-transcriptional regulation. Therefore, inducing TTP re-expression through epigenetic reprogramming or RNA interference is a key strategy to restore its tumor suppressor function.

#### 4.2.1. Transcriptional Derepression Mediated by DNA Demethylation

Aberrant promoter CpG island hypermethylation is a major mechanism causing TTP expression loss in multiple human cancers, particularly hepatocellular carcinoma and breast cancer. This epigenetic silencing relieves TTP post-transcriptional repression of oncogenes (e.g., c-Myc, Bcl-2), driving tumor progression. Tran et al. demonstrated that treatment of liver cancer cells with DNA methyltransferase inhibitors (DNMTi) such as 5-Aza-2′-deoxycytidine (Decitabine) effectively reverses methylation of the ZFP36 promoter [92]. The resulting demethylation reactivates ZFP36 transcription; restored TTP induces rapid tumor cell cycle arrest and apoptosis [92]. These findings suggest that for refractory tumors with complete or partial TTP silencing, combining epigenetic drugs (e.g., HDAC inhibitors, DNMT inhibitors) with standard regimens to restore TTP function is a promising strategy requiring further preclinical evaluation.

#### 4.2.2. Targeted Blockade of Non-Coding RNA Networks

Besides DNA methylation, microRNA (miRNA)-mediated post-transcriptional regulation is another major cause of TTP downregulation. In invasive breast cancer models, Gebeshuber et al. found aberrantly elevated miR-29a directly targets ZFP36 mRNA 3′UTR, promoting its degradation or translational repression, facilitating epithelial–mesenchymal transition (EMT) and tumor metastasis [88]. Therapeutic approaches employing antisense oligonucleotides (antagomirs) or miRNA sponges to specifically block miR-29a substantially restore endogenous TTP levels, reinstituting tumor invasive phenotype suppression [88]. This highlights RNA interference therapies as precise tools for reactivating RBP-mediated tumor suppressor networks.

#### 4.2.3. Epigenetic Regulation by Natural Compounds

In addition to synthetic drugs, certain dietary bioactive compounds (nutraceuticals) upregulate TTP via complex signaling networks, exerting anti-inflammatory and anti-cancer effects. For example, polyphenol resveratrol exhibits natural DNA methyltransferase (DNMT) inhibitory activity, blocking gene promoter epigenetic silencing and robustly inducing ZFP36 mRNA and protein expression in human glioma cells [124]. Endogenous TTP restoration reactivates urokinase plasminogen activator (uPA) and its receptor (uPAR) mRNA degradation, effectively inhibiting glioma cell growth and inducing apoptosis [124].

### 4.3. Synergistic Regulation Based on RNA-Binding Protein Networks

Other RBPs competitively influence TTP post-transcriptional regulation, notably HuR (ELAVL1), a direct antagonist. HuR also binds AREs in mRNA 3′UTRs but, unlike TTP deadenylation-complex recruitment, HuR binding stabilizes target mRNA and promotes translation. Consequently, TTP–HuR binding balance at shared targets sets transcript expression levels.

#### 4.3.1. Post-Transcriptional Competitive Antagonism Between TTP and HuR

TTP and HuR directly compete for binding to mRNAs of key oncogenic and inflammatory factors, including VEGF and COX-2. Via reciprocal antagonism, these proteins finely tune expression levels of critical targets. Under hypoxia or tumor microenvironment conditions, HuR affinity for VEGF mRNA elevates, stabilizing VEGF and promoting angiogenesis [85]. Conversely, TTP re-expression displaces HuR from COX-2 and MMP-9 mRNAs, reversing mRNA stability and blocking inflammatory cascades and tumor invasiveness [125,126]. TTP tumor-suppressive effects thus depend not only on expression level but also on HuR competitive dominance at oncogenic mRNA binding sites.

#### 4.3.2. Clinical Prognostic Value of the HuR/TTP Ratio

As TTP and HuR compete directly at the post-transcriptional level, single-molecule expression often fails to reflect their functional status in tumorigenesis. The HuR/TTP expression ratio has thus emerged as a biologically relevant and clinically predictive biomarker. In aggressive colorectal cancer, tumor tissues exhibit elevated HuR nuclear–cytoplasmic shuttling alongside silenced TTP expression, driving a high HuR/TTP ratio [67]. This imbalance correlates with shorter disease-free survival (DFS), higher tumor grade, and chemotherapy resistance [67]. Similar patterns in colon and gastric cancers indicate that an elevated HuR/TTP ratio drives malignant phenotype and represents a potential therapeutic target.

#### 4.3.3. Combined Targeted Therapy Strategies Based on the Antagonistic Relationship Between TTP and HuR

Given TTP–HuR competitive antagonism in ARE-mRNA stability regulation, combined TTP activators and HuR inhibitors may produce synergistic therapeutic effects. In monotherapy, small-molecule inhibitors (e.g., MS-444 or CMLD-2), which block HuR dimerization or nuclear–cytoplasmic shuttling, reduce HuR protection of oncogenic mRNAs [127,128]. Independently, DNA demethylating agents (e.g., decitabine) reverse epigenetic silencing, restoring endogenous TTP expression and tumor suppressor function [92]. As TTP and HuR compete for the same oncogenic mRNA targets, combination therapies blocking HuR while activating TTP may synergistically reshape RNA stability networks, overcoming single-target drug limitations and enabling precision medicine.

### 4.4. Safety and the Physiological-Pathological Paradox in Clinical Translation

Although enhancing ZFP36 family activity holds therapeutic promise in malignancy and chronic inflammation, these proteins exhibit complex regulatory characteristics in physiological tissue repair. In specific regenerative microenvironments, transient ZFP36 family member downregulation is necessary to initiate reparative programs. Nonspecific systemic activation strategies may thus interfere with normal wound healing or disrupt stem cell function.

Two further concerns merit consideration. First, as these proteins regulate broad inflammatory and proliferative transcript regulons, globally elevating their activity risks indiscriminately suppressing mRNAs required for normal immune defense and tissue turnover, rather than selectively correcting pathological ones. Second, the family operates within a redundant, competitive RBP network (Section 4.3): single-point intervention may trigger compensatory rebound (e.g., HuR upregulation or paralogous family member upregulation) that blunts or distorts the intended effect. These considerations favor context-restricted, network-aware approaches over simple “more ZFP36 activity is better” logic.

#### 4.4.1. Risk of Angiogenesis Inhibition During Tissue Repair

Effective skin wound healing requires rapid VEGF induction to promote angiogenesis, making ZFP36 family member downregulation a critical physiological adaptive mechanism. During wound healing, miR-93-3p is significantly upregulated in keratinocytes, targeting and suppressing ZFP36L1 expression [60], thus relieving repression of growth factors (e.g., ZFX and VEGF) [31,60]. ZFP36L1 functional inhibition maintains local growth factor levels and supports epithelial cell proliferation and migration. Forced ZFP36L1 activation during this period may excessively degrade VEGF mRNA, impairing angiogenesis and causing chronic ulcers or delayed wound closure [31,60].

#### 4.4.2. Balancing Organ Fibrosis and Acute Injury Repair

In visceral organ repair, ZFP36 exhibits a time-dependent, opposing role. In acute ischemia–reperfusion (I/R) injury models, ZFP36 expression limits excessive inflammation: ZFP36 suppresses inflammation and apoptosis via CREBBP mRNA degradation [70]. However, during chronic repair, ZFP36 functional loss in lung fibrosis models elevates IER3 mRNA stability, driving fibroblast hyperproliferation and abnormal collagen accumulation [129]. These findings indicate that ZFP36 therapeutic targeting requires phase-specific consideration: enhanced activity benefits acute inflammation resolution and organ protection, whereas persistent overexpression during tissue repair may block regeneration.

#### 4.4.3. Overcoming Systemic Toxicity: Potential of Spatiotemporal Precision Delivery Systems

Given ZFP36 family physiological and pathological functional complexity, broad systemic activation risks severe off-target toxicity. Although ZFP36 family-specific targeted delivery remains early-stage, spatiotemporally controllable precision delivery systems represent a key theoretical direction for overcoming clinical translation barriers.

To avoid local angiogenesis inhibition and delayed wound healing caused by systemic ZFP36L1 activation [60], future delivery systems could incorporate spatial specificity. Lipid nanoparticles (LNPs), promising in targeted delivery [130], could be engineered with pro-inflammatory macrophage receptor ligands (e.g., CD163) to selectively deliver TTP activators, suppressing local inflammation while sparing normal keratinocytes and preserving physiological VEGF expression [31,60]. Antibody–drug conjugates (ADCs) offer an additional avenue for precise ZFP36 modulation [131]: PP2A agonists relieving TTP phosphorylation suppression could be conjugated with tumor-specific antibodies (e.g., anti-EGFR), enabling drug release exclusively in the tumor microenvironment and remodeling tumor suppressive networks while minimizing toxicity to normal hematopoietic and physiological barriers [131].

Addressing the ZFP36 temporal paradox in acute injury defense versus chronic repair [70,129], stimuli-responsive nanocarriers provide a promising conceptual framework [132]. ROS- or pH-sensitive nanocarriers encapsulating TTP activators [132] could degrade under acute high-ROS inflammation to release drug, upregulating TTP to degrade CREBBP mRNA and quell inflammatory storms [70]. As ROS diminishes with disease progression, drug release ceases, allowing local TTP activity to return to physiological baseline and protecting normal fibroblast proliferation and collagen deposition, blocking fibrosis caused by persistent TTP overactivation [129].

ZFP36 modulation via advanced delivery technologies remains largely conceptual. If experimentally validated, these approaches could shift pharmacological interventions from systemic broad activation toward pathological microenvironment-driven activation, though substantial preclinical development and validation remain outstanding for ZFP36-targeted agents.

## 5. Summary, Limitations, and Future Perspectives

The ZFP36 family regulates gene expression post-transcriptionally. As critical trans-acting factors recognizing AREs, family members maintain immune homeostasis and restrain excessive tissue stress responses.

Under physiological conditions, the ZFP36 family regulates target mRNA degradation to establish innate immunity activation thresholds and maintain adaptive immune cell quiescence. Concurrently, these proteins are key homeostatic regulators of stem cell fate decisions and hematopoietic, nervous, and vascular system development [4,12,21]. This post-transcriptional regulatory network enables rapid, spatiotemporally specific gene expression adjustment in response to environmental cues.

Under pathological states, genetic alterations (e.g., TTP gene deletions), epigenetic modifications (e.g., promoter hypermethylation), or abnormal kinase signaling (e.g., p38 MAPK-driven hyperphosphorylation) impair ZFP36 family mRNA degradation function. The resulting prolonged half-lives and abnormal accumulation of target transcripts, including pro-inflammatory factors, chemokines, and oncogenes, directly contribute to chronic inflammation persistence, immune tolerance breakdown, and tumor invasion and metastasis [28]. TTP has also been implicated in mitochondrial dynamics, epigenetic–metabolic crosstalk, and blood–brain barrier integrity, linking post-transcriptional RNA metabolism to metabolic and multisystem disease [109,133].

Although ZFP36 family pharmacological activation shows promising intervention potential in preclinical models, clinical translation remains hindered by an oversimplified “binary” perception that categorizes ZFP36 merely as an “inflammatory brake” or a general tumor suppressor, overlooking its nonredundant and sometimes antagonistic roles in distinct cellular subpopulations. For instance, systemic activation may suppress inflammatory storms effectively but also inadvertently disrupt essential angiogenic signals required for normal tissue repair. Additionally, dynamic competition between ZFP36 and oncogenic RBPs (e.g., HuR) at shared mRNA targets renders nonspecific single-point interventions prone to complex compensatory rebounds.

Several limitations warrant explicit acknowledgment. First, disease-association data, particularly from clinical specimens, are largely correlative; causal ZFP36 dysregulation roles have been functionally established in only a subset of contexts. Second, high redundancy and potential compensation among TTP, ZFP36L1, and ZFP36L2 complicate single-gene perturbation study interpretation and therapeutic outcome prediction. Third, as these proteins regulate broad transcript networks, modulating their activity carries inherent off-target risk to physiological immune and reparative processes. Fourth, tissue- and cell-type-specific ZFP36 modulator delivery remains technically unsolved, with no clinically validated platform currently existing for this purpose. Fifth, reliable biomarkers capturing ZFP36 functional status (as opposed to expression level alone) are lacking, which is essential given functionally silenced but abundant TTP (Section 3.2.1). How TTP phosphorylation coordinates stress granule and P-body partitioning, and how this integrates with cellular stress adaptation, remains an open mechanistic question unresolved by current models. Recognizing these limitations is a prerequisite for translating ZFP36 biology into rational therapeutic strategies.

Future ZFP36 family research should move beyond macroscopic phenotype observation toward precise dissection of microenvironment–target network interactions. At the basic level, spatial transcriptomics and CLIP-seq could map dynamic ZFP36 and paralog (ZFP36L1/L2) interaction networks at single-cell resolution across disease stages. Clinically, effective targeting will require spatial and temporal precision beyond specific small molecules. Microenvironment-responsive carriers or cell-specific delivery systems (e.g., targeted LNPs) may enable precise correction of dysregulated post-transcriptional networks at critical disease nodes, potentially separating therapeutic anti-inflammatory and tumor-suppressive effects from normal homeostasis disruption.

Translating ZFP36 biology into therapy requires resolving context-specific functions and closing the mechanistic and translational gaps outlined above: distinguishing functionally silenced from genuinely downregulated protein, and modulating individual family members in a cell-type-specific manner without disrupting physiological homeostasis.

## Figures and Tables

**Table 2:** Summary of the functions of the ZFP36 family in physiological development and homeostasis maintenance.

System	Cell Type	Members	Representative Target/Mechanism	Principal Function	Ev.	Ref
Innate Immunity	Macrophages	TTP	Degrades TNF, IL-6; inhibits NLRP3	Sets inflammatory threshold; promotes resolution	F	[37,38,39,40,41,42]
	Dendritic cells	TTP	Degrades IL-23α	Prevents aberrant DC maturation	F	[43,44,45]
Adaptive Immunity	Conv. T cells	TTP	Degrades IL-2, IFN-γ	Restrains T-cell activation	F	[46,47,48]
	Th17 cells	TTP	Degrades IL-17α	Limits Th17 pathogenicity	F	[49]
	CD8^+^ T cells	L1, L2	L1 restrains early differentiation (NF-κB); L2 represses IFN-γ	Time-restrict cytotoxic differentiation	F	[50,51]
	Memory T cells	L2	Stores IFN-γ mRNA	Enables rapid recall response	F	[35]
	Treg cells	L1, L2	Degrades Helios (Ikzf2)	Maintains Treg suppressive function	F	[36,52]
	B cells	L1, L2	Degrades Ccnb1/Cdk1	Enforces G2-M checkpoint	F	[32,33,53]
Barriers & Repair	Gut epithelium	TTP	Degrades Nos2	Maintains mucosal homeostasis	F	[54]
	Airway epithelium	TTP, L1, L2	Degrades IL-6, IL-8	Limits neutrophilic lung inflammation	F	[55,56,57,58]
	Keratinocytes	TTP, L1	L1 represses VEGF	Restrains skin inflammation; downregulated upon injury for repair	F	[31,49,59,60]
Stem Cells & Dev.	HSC	L2	Suppresses mTORC1	Maintains HSC quiescence	F	[12,61,62]
	Lymphoid progenitors	L1, L2	Restrict Notch1	Prevent T-ALL transformation	F	[4,11]
	Erythroid progenitors	L1	Stat5b	Restrains erythroid over-proliferation	F	[13,34]

Note. Members: L1 = ZFP36L1, L2 = ZFP36L2. Ev. (Evidence): F = functional (gene knockout/knockdown or in vitro mechanistic study). Only representative directly validated targets are listed; additional targets are described in the main text.

**Table 3:** Summary of the Pathological Roles of the ZFP36 Family in Human Diseases.

Disease Category	Specific Disease	Member	Expression/Status	Representative Target/Mechanism	Pathological Association	Ev.	Ref
Malignant Tumors	Breast cancer	TTP	Downregulated (miR-29a)	Derepression of AP-1/c-Jun	Cell-cycle progression and EMT	F	[80,88,89,90]
	Colorectal cancer	TTP	Silenced/high HuR/TTP ratio	Stabilization of COX-2, VEGFA	Inflammatory microenvironment, angiogenesis, chemoresistance	F/O	[67,85,91]
	Head and neck SCC	TTP	Silenced	Stabilization of MMP2/9, CD47	Invasion, metastasis, immune evasion (CD47/SIRPα)	F	[81,87]
	Hepatocellular carcinoma	TTP	Promoter methylation	Derepression of c-Myc, Snail1	EMT and tumor progression	F	[83,92]
	B-cell malignancies	TTP, L1	Impaired	Stabilization of c-Myc, BCL2	Lymphomagenesis, abnormal survival	F	[53,65,66]
	Solid tumor hypoxia	TTP	Lost	Stabilization of HIF-1α	Glycolytic reprogramming, angiogenesis	F	[84,85,86]
	Bladder cancer (MIBC)	L1	Context-dependent (↑ advanced stage)	Associated with EMT-pathway activation	Enhanced invasion at advanced stage (stage-dependent role)	O	[93]
	Pancreatic ductal adenocarcinoma	L2	Conflicting reports	Oncogenic claim (↑, miR-375 loss) [94] vs. loss-of-function tumor suppressor [95]	Unresolved controversy (oncogenic vs. tumor-suppressive)	F (conflicting)	[94,95]
	Ovarian cancer (mesenchymal)	TTP/HuR	TTP low; HuR-dominated	HuR/TTP imbalance at shared AREs; TTP restoration degrades Twist1/Snail1 (Section 4.3)	EMT, invasion	F/O	[96,97,98]
Autoimmunity & Inflammation	Rheumatoid arthritis	TTP	p38/MK2 hyperphosphorylation (functional silencing)	Stabilization of TNF-α, IL-6	Persistent synovial inflammation	F	[16,17,29,31,38,67,99,100]
	Inflammatory bowel disease	TTP	Inactivation/impaired	Stabilization of IL-23α, IL-17α	IL-23/IL-17 axis, barrier disruption	F/O	[44,49,101]
	Psoriasis	TTP	Promoter methylation	NLRP3 inflammasome activation	Spontaneous skin inflammation	F/O	[102]
Cardiovascular	Vascular restenosis	L1	Downregulated	Derepression of KLF16	VSMC phenotype switch, neointimal hyperplasia	F	[103,104]
	Vascular calcification	TTP	Upregulated	Degradation of Runx2	Protective: limits VSMC osteogenic transdifferentiation	F	[105]
	Hypertension	TTP	Upregulated in Ang II models	Degradation of Rgs2	Associated with enhanced vasoconstriction in experimental hypertension	O	[106,107,108]
Nervous System	Cerebral I/R injury	TTP	Impaired	Stabilization of Nox4, Drp1	Mitochondrial fission, ROS, neuronal apoptosis	F	[109]
	Parkinson’s disease	TTP	Impaired	Stabilization of NOX2	Oxidative stress in dopaminergic neurons	F	[110]
	Alzheimer’s disease	TTP	↑ (bidirectional dysregulation)	↑TTP degrades BDNF (harmful); compensatory TTP degradation of ZBP1 (protective)	Reduced neurotrophic support; microglial inflammation	F/O	[111,112,113]

Note. Member: L1 = ZFP36L1, L2 = ZFP36L2. Ev. (Evidence basis): F = functional (gene perturbation or in vitro mechanistic study); O = observational (expression/correlative analysis of clinical specimens); F/O = both. Entries labeled “O”—including the bladder (MIBC), pancreatic, ovarian cancer, hypertension, and Alzheimer’s disease entries—reflect associations for which causal mechanisms require further functional validation. Context-dependent entries denote settings in which the family member’s role diverges from its canonical tumor-suppressive function (see Section 3.1.3). For entries with multiple cited targets, additional targets and mechanisms are described in the main text. In this table, ↑ indicates upregulation.

## Data Availability

No new data were created or analyzed in this study.

## Abbreviations

The following abbreviations are used in this manuscript:

AAL(S)	2-Amino-2-methyl-4-(4-octylphenyl) butan-1-ol
AD	Alzheimer’s disease
ADCs	Antibody–drug conjugates
Akt/PKB	Protein kinase B
ALI	Acute lung injury
Ang II	Angiotensin II
AP-1	Activator protein 1
ARE	AU-rich element
Areg	Amphiregulin
BCL2	B-cell lymphoma 2
BDNF	Brain-derived neurotrophic factor
BLIMP1	B lymphocyte-induced maturation protein-1
CCL3	C-C motif chemokine ligand 3
CCNB1	Cyclin B1
CCR4-NOT	Carbon catabolite repressor 4–negative on TATA-less
CD	Cluster of differentiation
CDK1	Cyclin-dependent kinase 1
CIM	CNOT1-binding motif
CLIP-seq	Crosslinking immunoprecipitation sequencing
CNOT1	CCR4-NOT transcription complex subunit 1
COPD	Chronic obstructive pulmonary disease
COX-2	Cyclooxygenase-2
CREBBP	CREB binding protein
CRM1	Chromosome region maintenance 1
CTRP13	C1q/TNF-related protein 13
CXCL	C-X-C motif chemokine ligand
DC	Dendritic cell
DCP1/2	Decapping mRNA 1/2
DFS	Disease-free survival
DNMT	DNA methyltransferase
DNMTi	DNA methyltransferase inhibitor
DRP1	Dynamin-related protein 1
DUSP1	Dual-specificity phosphatase 1
ECM	Extracellular matrix
EGFR	Epidermal growth factor receptor
EGR1	Early growth response protein 1
ELAVL1	ELAV-like RNA binding protein 1
EMT	Epithelial–mesenchymal transition
eNOS	Endothelial nitric oxide synthase
ERAD	Endoplasmic reticulum-associated degradation
ERK	Extracellular signal-regulated kinase
FOXM1	Forkhead box M1
FTY720	Fingolimod
G2-M	G2-M cell cycle checkpoint
GYF2	GYF domain-containing protein 2
HDAC	Histone deacetylase
HIF-1α	Hypoxia-inducible factor 1-alpha
HLA-DQ	Human leukocyte antigen-DQ isotype
HSC	Hematopoietic stem cell
HuR	Human antigen R
IBD	Inflammatory bowel disease
ICAM-1	Intercellular adhesion molecule-1
IDO1	Indoleamine 2,3-dioxygenase 1
IDR	Intrinsically disordered region
IEC	Intestinal epithelial cell
IER3	Immediate early response 3
IFN-γ	Interferon gamma
Ikzf2	IKAROS family zinc finger 2
IL	Interleukin
iNOS	Inducible nitric oxide synthase
I/R	Ischemia–reperfusion
IRF8	Interferon regulatory factor 8
KLF	Krüppel-like factor
KYNA	Kynurenic acid
lncOlfr29	Long noncoding RNA Olfr29
LNP	Lipid nanoparticle
MAPK	Mitogen-activated protein kinase
MDSC	Myeloid-derived suppressor cell
MIBC	Muscle-invasive bladder cancer
miRNA/miR	MicroRNA
MK2	MAPK-activated protein kinase 2
MKP-1	Mitogen-activated protein kinase phosphatase 1
MMP	Matrix metalloproteinase
mRNA	Messenger RNA
mTORC1	Mammalian target of rapamycin complex 1
MZ	Marginal zone
ncRNA	Non-coding RNA
NES	Nuclear export signal
NF-κB	Nuclear factor kappa B
NLRP3	NLR family pyrin domain containing 3
NO	Nitric oxide
NOX	NADPH oxidase
NSC	Neural stem cell
OVC	Ovarian cancer
P-bodies	Processing bodies
PD	Parkinson’s disease
PDAC	Pancreatic ductal adenocarcinoma
PI3K	Phosphoinositide 3-kinase
PLAU (uPA)	Plasminogen activator, urokinase
PLAUR (uPAR)	Plasminogen activator, urokinase receptor
PP2A	Protein phosphatase 2A
PRDM1	PR domain zinc finger protein 1
RA	Rheumatoid arthritis
RALDH2	Retinaldehyde dehydrogenase 2
RBP	RNA-binding protein
RGS2	Regulator of G protein signaling 2
ROQUIN	RC3H1, Roquin-1
ROS	Reactive oxygen species
RSK	Ribosomal s6 kinase
RUNX2	RUNX family transcription factor 2
SCC	Squamous cell carcinoma
SIRPα	Signal regulatory protein alpha
α-SMA	Alpha-smooth muscle actin
SM-MHC	Smooth muscle myosin heavy chain
SNAIL1	Snail family transcriptional repressor 1
SOCS7	Suppressor of cytokine signaling 7
STAT5b	Signal transducer and activator of transcription 5b
T-ALL	T-cell acute lymphoblastic leukemia
TCR	T-cell receptor
Th17	T helper 17 cell
TME	Tumor microenvironment
TNF-α/Tnf	Tumor necrosis factor-alpha
Treg	Regulatory T cell
TTP	Tristetraprolin
TZF	Tandem CCCH zinc finger domain
uPA	Urokinase plasminogen activator
uPAR	Urokinase plasminogen activator receptor
3′-UTR	3′ untranslated region
VCAM-1	Vascular cell adhesion molecule-1
VEGF/VEGFA/Vegf(a)	Vascular endothelial growth factor (A). Note: lowercase “Vegf/Vegfa” follows mouse gene nomenclature; “VEGF/VEGFA” follows human/general nomenclature. Both refer to the same molecular family.
VSMC	Vascular smooth muscle cell
XRN1	5′-3′ exoribonuclease 1
ZBP1	Z-DNA binding protein 1
ZFP36	Zinc finger protein 36
ZFX	Zinc finger protein X-linked
